# Editorial: Resistance and Tolerance in Food-Borne Pathogens: Mechanisms, Public Health Impact, and Control Measures

**DOI:** 10.3389/fmicb.2021.769931

**Published:** 2021-10-14

**Authors:** Byeonghwa Jeon, Taradon Luangtongkum, Zhangqi Shen, Catherine M. Logue, Jun Lin

**Affiliations:** ^1^Division of Environmental Health Sciences, School of Public Health, University of Minnesota, Minneapolis, MN, United States; ^2^Department of Veterinary Public Health, Faculty of Veterinary Science, Chulalongkorn University, Bangkok, Thailand; ^3^Beijing Key Laboratory of Detection Technology for Animal-Derived Food Safety, Beijing Laboratory of Food Quality and Safety, College of Veterinary Medicine, China Agricultural University, Beijing, China; ^4^Department of Population Health, College of Veterinary Medicine, University of Georgia, Athens, GA, United States; ^5^Department of Animal Science, University of Tennessee, Knoxville, Knoxville, TN, United States

**Keywords:** foodborne pathogens, food safety, public health, antibiotic resistance, stress tolerance

Foodborne pathogens are exposed to many stress conditions present in animal hosts and the environment surrounding farms at the stage of primary production and encounter artificial stressors, including disinfectants and non-optimal growth conditions, during food processing, storage, and cooking. Foodborne pathogens can cause foodborne illnesses only when they successfully overcome stress conditions along the farm to fork continuum (Begley and Hill, 2015; Oh et al., 2018; Zhang F. et al.). Most foodborne infections are self-limiting; however, serious cases of illness require antimicrobial chemotherapy. With the increasing prevalence of antibiotic-resistant foodborne pathogens being detected, the efficacy of antimicrobial chemotherapy is significantly compromised, leading to serious patient outcomes. Bacterial tolerance to environmental stress and resistance to antibiotics significantly affect food safety and public health. Although stress tolerance and antibiotic resistance in bacteria may be considered totally different topics, there are some overarching features for both. For instance, oxidative stress is a general tolerance mechanism that affects the lethality of antibiotic treatment (Poole, 2012). Thus, understanding stress tolerance and antibiotic resistance in foodborne pathogens can provide a holistic perspective for food safety from farm to fork and beyond, including clinical treatment of foodborne illnesses. The Research Topic “*Resistance and Tolerance in Food-borne Pathogens: Mechanisms, Public Health Impact, and Control Measures*” aims to extend our knowledge of how these mechanisms influence food safety and public health.

Two articles about stress tolerance in *Listeria monocytogenes* were published. Zhang H. et al. reported that *L. monocytogenes* 1/2b isolates were the predominant strain types isolated from processing facilities of ready-to-eat meat in China irrespective of the observed hygiene levels based on aerobic plate counts and coliform detection. Whole genome sequence analysis suggested that the isolates clonally expanded possibly by forming biofilms, confirming the importance of sanitation procedures for the control of *L. monocytogenes*. Interestingly, the findings of Li et al. showed that the use of organic acids increases the transcriptional levels of genes associated with acid and bile stress response and virulence. Exposure to acetic acid and lactic acid, both common organic acids used in the food industry, increase the virulence of *L. monocytogenes* based on testing using the *Galleria mellonella* infection model. Organic acids are frequently used for the control of *Listeria* contamination by the food industry. However, organic acids can potentially influence *Listeria's* stress tolerance and virulence.

With respect to various *in vivo* stresses encountered by microbes, foodborne pathogens also rely on effective and complex tolerance strategies to survive and establish successful infection in the host. One such study is focused on the resistance of *Vibrio vulnificus* to nitric oxide (NO), an important antimicrobial effector produced by the host innate immune system to counteract invading pathogens (Choi et al.). The investigators identified a NO-responsive transcriptional regulator NsrR, a strong repressor of *hmpA* that encodes an NO dioxygenase required for resistance of *V. vulnificus* to nitrosative stress. Further molecular studies found that NsrR could delicately cooperate with other two regulatory proteins, Lrp and CRP, to tightly control the transcription of *hmpA*, consequently contributing to the survival of *V. vulnificus* under host-derived nitrosative stress (Choi et al.).

Several articles in the eCollection ascertained that antibiotic resistance is highly common in the food supply chain worldwide. The article by Lopez-Chavarrias et al. provides a good demonstration of the high prevalence of antibiotic-resistant *Campylobacter* in healthy livestock in Spain (Lopez-Chavarrias et al.). Approximately 94.5% of *Campylobacter coli* isolates and 91.1% of *Campylobacter jejuni* isolates from broilers were resistant to ciprofloxacin, a fluoroquinolone drug of clinical importance in human health, and 66.6% of *C. coli* from pigs were resistant to erythromycin. Consistently, tetracycline and fluoroquinolone resistance are prevalent in *C. jejuni* isolates from patients in the United States. Notably, campylobacteriosis associated with fluoroquinolone resistance was significantly associated with international travel (Rodrigues et al.). Barbieri et al. reported that *mcr-1* is highly prevalent in *Escherichia coli* isolates from healthy and sick poultry with colibacillosis in Brazil due to the agricultural application of colistin, a last-resort antibiotic to treat Gram-negative infections (Liu et al., 2016). In addition, multi-drug resistance in *Yersinia enterocolitica* 4/O:3 derived from fresh pre-washed spinach was found to be the cause of the consecutive foodborne yersiniosis outbreaks in Sweden in 2019. Molecular characterization of the multidrug-resistant *Y. enterocolitica* outbreak strain revealed that this foodborne pathogen harbored the Tn*2670* transposon with resistance determinants against quaternary ammonium compounds, the heavy metal mercury, phenicols, streptomycin, and sulfonamides and an additional plasmid carrying tetracycline resistance gene. Interestingly, neither the Tn*2670* transposon nor the *tet*B resistance plasmid has previously been reported in foodborne *Yersinia* nor in isolates derived from ready-to-eat products, suggesting that horizontal gene transfer events occurring in the environment, agriculture, or animal husbandry have promoted the selection of *Y. enterocolitica* carrying multi-antibiotic and metal resistance determinants (Karlsson et al.).

An article included in this collection discussed potential intervention measures to control antibiotic resistance in the food supply chain using bacteriophages (phages). Kim et al. discovered that some phages preferentially infect *E. coli* based on the phylogenetic group and constructed a highly effective phage cocktail targeting poultry isolates of *E. coli* producing extended-spectrum β-lactamases (ESBL) that frequently contaminate retail poultry. Although antibiotic-resistant, non-pathogenic *E. coli* does not develop an infection in animals and humans, antibiotic resistance can be transferred to pathogenic bacteria. The phages strongly inhibited ESBL-producing *E. coli* on chicken skin at refrigeration temperatures, suggesting that phages have potential application for use in retail raw chicken to reduce antibiotic resistance (Kim et al.).

Humans are continuously exposed to antibiotic-resistant microorganisms through the consumption of food, and the chances of exposure to antibiotic-resistant microorganisms will continue to pose a challenge and potentially increase in prevalence if foodborne pathogenic bacteria can survive stress conditions in the pathway from farm to fork. These articles highlight the concern about food chain contamination as a potential reservoir for transmission and dissemination of antimicrobial resistance, raising concerns for food safety and public health.

## Author Contributions

All authors listed have made a substantial, direct and intellectual contribution to the work, and approved it for publication.

## Conflict of Interest

The authors declare that the research was conducted in the absence of any commercial or financial relationships that could be construed as a potential conflict of interest.

## Publisher's Note

All claims expressed in this article are solely those of the authors and do not necessarily represent those of their affiliated organizations, or those of the publisher, the editors and the reviewers. Any product that may be evaluated in this article, or claim that may be made by its manufacturer, is not guaranteed or endorsed by the publisher.
